# Assessment Disparities among Pediatric Patients: Advantages of Pictorial Descriptions

**DOI:** 10.3389/fped.2013.00028

**Published:** 2013-10-15

**Authors:** Marie Leiner, Jesus Peinado, Maria Theresa Malazo Villanos, Patricia Jimenez

**Affiliations:** ^1^Department of Pediatrics, Texas Tech University Health SciencesEl Paso, TX, USA; ^2^Department of Social Sciences and Administration, Universidad Autonoma de Ciudad Juarez, Ciudad JuarezChihuahua, Mexico

**Keywords:** assessment disparities, pediatric patients, pictorial descriptions, health literacy, health disparities

Remarkable progress in medicine has provided people with the potential for longer and healthier lives. However, health disparities (e.g., poverty, access to health care, educational inequalities, higher exposure to health risks, etc.), make individuals, families, and communities miss most of the benefits of this progress (1, 2). Pediatric populations are affected by these disparities during crucial years of development. The result is poor health or disease, which develops over short and/or long term periods (3–5). The adverse contributions of these disparities have a different effect on each individual. However, they have a powerful effect on pediatric outcomes. One of the most recognized factors associated with health disparities is the problem of poor communication with parents/caretakers. Poor communication prevents them from receiving health benefits, participating in research studies, and actively contributing to disease prevention and health care for their children (6–9). Decades of effort and research on strategies to reduce health disparities have led to inconclusive and conflicting results, and there has been little improvement in leading health indicators (10). Some interventions could have a true beneficial effect on the participants. However, it is possible that the findings have been affected by misclassification of outcomes, exposures, or health conditions due to assessment disparities (11–13).

Research, screening, and diagnosis of pediatric health problems rely on the parents/caretakers ability to understand and respond to health questionnaires and other assessment tools. The accuracy and precision of the results might be compromised when the parents respond to written consent forms, questionnaires, and/or screening tools (14). The root of this communication problem has been attributed to disparities caused by lower levels of education, lack of language proficiency, low literacy, and cultural differences (15, 16).

It is possible that, due to these communication problems, we are missing a critical component of measurement because *assessment disparities* prevent us from substantially measuring baseline and follow up indicators simply because the assessments used are not understood. The result is inaccurate data. This issue might contribute to differences between and within an ethnic group and affect the final results of interventions, planning health needs, and early interventions. Therefore, it is essential that we include measures that allow us to increase our precision and discover the real needs, status, and results of interventions among those that are confronting disparities. This approach can benefit our knowledge of groups affected by heath disparities through increasing their participation in research studies, which is preferable to simply looking for associations that lead to better health outcomes (e.g., Hispanic paradox, healthy migrant effect, etc.) (17–22).

Strategies to increase research participation and funding have been proposed by various institutions (e.g., the National Institute on Minority Health and Health Disparities in the United States and the World Health Organization) (23). The central theme has included innovative methods of transmitting information (24–28); for example, oral and/or video instructions combined with written material could increase understanding of the information (29–32). These strategies have proven effective at increasing communication with participants however, study accuracy can still be compromised when studies include written-format questions, requiring the participant to read and write (33–35). When the participant is unable to read and/or write, the questions are often read aloud by the investigator, which can introduce bias and confounding factors. For example, a questionnaire with validity and reliability that was tested as a written instrument might not have the same validity when the responses are obtained in another way.

Pictorials have been used for communication since ancestral times. Pictorial descriptions with written instructions enhance understanding of questions for parents confronting literacy barriers and have proven value in cross-cultural communication of information (36, 37). Early studies in psychology and other sciences have demonstrated that pictorials increase cross-cultural communication because they can be used to explain procedures with few images. Pictorial representations can effectively convey simple sentences across language barriers because people share almost the same ability to understand the content of pictures (38–40).

**Figure 1:**
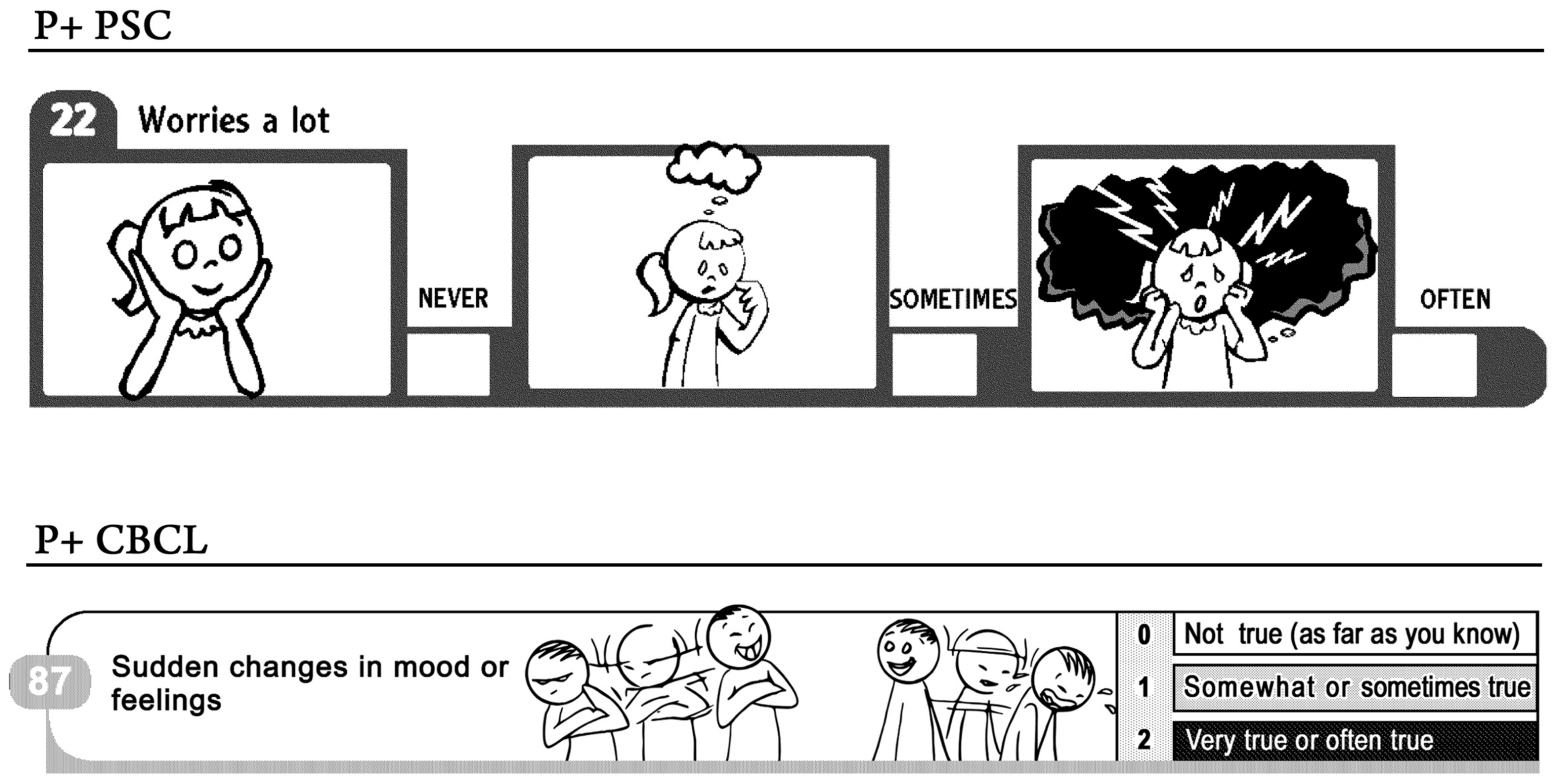
**Pictorial sample of questions from Pediatric Symptom Checklist (PSC) and Child Behavior Checklist (CBCL)**.

Our experience with pictorial descriptions has focused on mental health assessments. We found that adding pictorial descriptions to psychosocial and behavioral assessment tools increased detection and understanding of the questions without a need to change the content. We selected pictorials after unsuccessfully attempting to use other visual communication methods, because the pictorials were more effective at connecting actions and events (40). Pictorial development was complicated because our intention was to develop pictorials that would be used by different groups without depicting a specific ethnic group. Additionally, the pictorials needed to support the questions without suggesting different meanings, and finally, they needed to be user-friendly images instead of still, complicated images (see Figure 1). Pictorial descriptions reduce the complexity of the questions by mapping words to pictures. Compared with separate verbal and visual explanations, this approach promotes more effective creative problem solving (41, 42). A learner can construct and coordinate visual and verbal representations of the material, which leads to meaningful understanding of the written content.

Our first pictorial instrument included the Pediatric Symptom Checklist (43), which the Academy of Pediatrics recommends should be used to detect cognitive, emotional, and behavioral problems in children 4–16 years in age. We developed a version of the original instrument by adding pictorial descriptions to each of the 35 questions. We tested the results in participants confronting disparities (e.g., living in poverty, having lower health literacy) in the United States and Mexico. Compared with the original written instrument, the participant prevalence rate was more than double (44, 45). The next pictorial instrument we developed included the Child behavior Checklist (CBCL), which is the most used and recognized tool available for analyzing children with potential emotional and behavioral issues (46, 47).

The results of our study indicated that, when used for English and Spanish populations with low levels of literacy, the pictorial CBCL (P + CBCL) was equivalent to the written instrument (48). Pictorial descriptions added to the original CBCL helped parents confronting disparities to respond to the original questions in the survey instrument.

In our studies, we found that detection was improved by adding pictorial descriptions to a screening tool (44, 45, 49). Most importantly, the pictorial questionnaire produced an equivalent assessment when compared to the original questionnaire (48).

The advantageous characteristics of pictorial descriptions demonstrated in these studies should help increase their use and lead to benefits in a variety of areas. The use of pictorial descriptions in preventative material and other types of health communication may reduce communication gaps. Consent procedures could be simplified by the use of pictorial descriptions, which would increase research participation by improving understanding about the benefits of research studies (50, 51). For baseline indicator data collected via assessments or questionnaires, pictorial descriptions are very cost-effective, because the original assessments are not modified. Customizing a validated questionnaire to apply it to a specific population often requires extensive time and resources to change it, and to assess its validity and reliability. After the questionnaire is created, tested, and used in a population, the results are often not comparable to the results from the original questionnaire. In addition, the revised questionnaire might be applicable only to a specific population, so changes, and validation, for other populations are usually necessary.

Pictorial adaptations to other validated screening tools may facilitate greater use of these tools, with only a low added cost and reduce assessment disparities. A large number of subgroups worldwide would benefit, including those that encounter health disparities. The use of pictorial descriptions is complex. However, the benefits they can provide for measuring the impact of strategies and to increase communication between patient-providers lead us to conclude that their use is worth the effort.
